# IL-8 mediates idiopathic pulmonary fibrosis mesenchymal progenitor cell fibrogenicity

**DOI:** 10.1152/ajplung.00200.2017

**Published:** 2017-08-31

**Authors:** Libang Yang, Jeremy Herrera, Adam Gilbertsen, Hong Xia, Karen Smith, Alexey Benyumov, Peter B. Bitterman, Craig A. Henke

**Affiliations:** Department of Medicine, University of Minnesota, Minneapolis, Minnesota

**Keywords:** IPF mesenchymal progenitor cell, IL-8, macrophage, fibrotic front

## Abstract

Idiopathic pulmonary fibrosis (IPF) is a progressive fibrotic lung disease, but the mechanisms driving progression remain incompletely defined. We previously reported that the IPF lung harbors fibrogenic mesenchymal progenitor cells (MPCs), which serve as a cell of origin for IPF fibroblasts. Proliferating IPF MPCs are located at the periphery of fibroblastic foci in an active cellular front at the interface between the myofibroblast-rich focus core and adjacent normal alveolar structures. Among a large set of genes that distinguish IPF MPCs from their control counterparts, we identified IL-8 as a candidate mediator of IPF MPC fibrogenicity and driver of fibrotic progression. IPF MPCs and their progeny displayed increased steady-state levels of IL-8 and its cognate receptor CXCR1 and secreted more IL-8 than did controls. IL-8 functioned in an autocrine manner promoting IPF MPC self-renewal and the proliferation and motility of IPF MPC progeny. Secreted IL-8 also functioned in a paracrine manner stimulating macrophage migration. Analysis of IPF lung tissue demonstrated codistribution of IPF MPCs with activated macrophages in the active cellular front of the fibroblastic focus. These findings indicate that IPF MPC-derived IL-8 is capable of expanding the mesenchymal cell population and recruiting activated macrophages cells to actively evolving fibrotic lesions.

## INTRODUCTION

Idiopathic pulmonary fibrosis (IPF) progresses in a stereotypic manner. The disease begins in the peripheral basilar portions of the lungs (19, 28, 29). This nonrandom peripheral-basilar distribution of fibrosis gives rise to a chest computed tomography scan pattern now considered diagnostic for IPF (28). As the disease progresses, fibrosis spreads centrally in an axial distribution producing the distinct anatomical pattern designated usual interstitial pneumonia (27). Fibroblastic foci, the signature morphologic lesions of IPF, are frequently found at the leading edge of fibrosis (10, 16, 17, 23, 27). They vary greatly in size, ranging from small discrete lesions to large serpiginous structures (16). Our recent work suggests that there is temporal growth of fibroblastic foci as the lesions evolve due to local invasion of the fibrotic front into adjacent normal alveolar structures (42).

The molecular mechanism(s) driving fibrotic progression in IPF are just beginning to be elucidated. We previously reported the presence of fibrogenic MPCs in the IPF lung that serve as a cell of origin for the myofibroblasts mediating fibrotic destruction of the gas exchange apparatus (40, 41). IPF MPCs undergo robust self-renewal and manifest a durable fibrotic phenotype such that the progeny of a single MPC produces exuberant fibrotic lesions in xenograft models (40). Recently we performed immunohistochemical studies to delineate the number and location of IPF MPCs in fibroblastic foci. We found that the fibroblastic focus is a polarized structure. IPF MPCs and their early generation transit amplifying daughter cells are located at the periphery of the focus in a highly cellular interface region between the focus core containing mature myofibroblasts and more normal adjacent alveolar structures (42). This finding is in accord with the idea that this interface region represents an active, invasive fibrotic front (42).

We previously analyzed the transcriptome of IPF MPCs as they differentiate to disease-mediating fibroblasts (40, 42). Among a large set of genes that distinguish IPF MPCs from their control counterparts, we identified IL-8, a candidate mediator of IPF MPC fibrogenicity and driver of fibrotic progression (40). IL-8 (CXCL-8) is produced by a variety of immune and nonimmune cells including mesenchymal cells (4, 15, 35, 39). It belongs to the CXC family of small soluble proteins and was originally identified as a neutrophil chemoattractant (34). IL-8 biological effects are mediated through its binding to receptors CXCR1/2 (14). In addition to serving as a chemoattractant for immune cells, IL-8 promotes cell proliferation, motility, invasion, and epithelial-mesenchymal transition and has proangiogenesis functions (11, 20, 21, 36, 37, 43). Interestingly, recent studies have found IL-8 promotes cancer progression and regulates cancer stem cell activity via signaling through CXCR1 and CXCR2 (9, 38). Here we examined the role IL-8 plays in regulating the function of IPF MPCs and their progeny. We show that IL-8 is highly expressed and actively secreted by IPF MPCs and their progeny. It functions in an autocrine manner promoting the self-renewal of IPF MPCs and the proliferation and motility of IPF MPC progeny. It also functions in a paracrine manner stimulating the motility of macrophages. In situ analysis of IPF pathological specimens demonstrates that IPF MPCs codistribute with activated macrophages at the periphery of the fibroblastic focus in the highly cellular interface region between the myofibroblast core and adjacent more normal alveolar structures. These data indicate that IL-8 secreted by IPF MPCs and their progeny can serve the dual function of expanding the mesenchymal cell population and recruiting immunomodulatory cells to sites of evolving fibrotic lesions.

## METHODS

### 

#### Study approval.

Deidentified patient samples were obtained under a waiver of informed consent from the University of Minnesota Institutional Review Board.

#### Primary mesenchymal cell lines.

Five primary lung mesenchymal cell lines were established from patients fulfilling diagnostic criteria for IPF including a pathological diagnosis of usual interstitial pneumonia (3). Patient controls with nonfibrotic lung disorders were selected to be similar in age to IPF patients. Based on these criteria, we utilized seven nonfibrotic primary control mesenchymal cell lines established from lung tissue uninvolved by the primary disease process: squamous cell carcinoma (*n* = 1), adenocarcinoma (*n* = 4), as well as nonfibrotic control lung mesenchymal cell lines from one patient with emphysema and histologically normal lung tissue from a gunshot victim. Cell lines were derived from lungs, characterized as mesenchymal cells, and cultivated as previously described (40).

#### Isolation of MPCs.

IPF and control MPCs were isolated from primary IPF and control mesenchymal cell cultures at passage 0 (initial isolate before subcultivation) through passage 4. To isolate progenitors, primary IPF and control mesenchymal cells were labeled with mouse anti-human SSEA4 antibody conjugated to Alexa Fluor 647 and mouse anti-human SSEA1 conjugated to PE (BD Biosciences) as previously described (40, 42). Cells were sorted on a FACSAria Cell Sorter (BD Biosciences). Cells that were SSEA4^hi^ and SSEA1^−^ (relative to mouse IgG3 κ-isotype control conjugated to Alexa Fluor 647 and mouse IgM κ-isotype control conjugated to PE, respectively) and <12 μm were collected. To generate IPF and control MPC progeny, the SSEA4^hi^ MPCs were cultured in DMEM + 10% FCS for 21 days before use (hereafter designated IPF and control MPC progeny).

#### Quantitative-PCR.

Validity testing of IL-8 gene expression was conducted by quantitative (Q)-PCR. Total RNA was isolated and reverse transcribed using a Taqman Reverse Transcriptase Reagent Kit (Roche) and primed with random hexamers. Primer sequences were selected using National Center for Biotechnology Information Primer-BLAST. Real-time PCR (Q-PCR) was performed using a LightCycler FastStart DNA Master^PLUS^ SYBR Green I Kit (Roche). Primer sequences were as follows: IL-8 forward: 5′-CTTGGCAGCCTTCCTGA-3′ and IL-8 reverse: 5′- TTCTTTAGCACTCCTTGGCAAAA-3′; and CXCR1 forward: 5′-TGGGGACTGTCTATGAATCTGT-3′ and CXCR1 reverse: 5′-GCAACACCATCCGCCATTTT-3′. Samples were quantified at the log-linear portion of the curve using LightCycler analysis software and compared with an external calibration standard curve.

#### IL-8 ELISA.

IPF and control MPCs and their progeny were seeded in tissue culture dishes containing DMEM + 10% FBS and incubated for 48 h. IL-8 protein levels in cell lysates and culture medium were quantified using an IL-8 ELISA Kit according to the manufacturer’s instructions (R&D Systems, Minneapolis, MN). The concentration of IL-8 in culture medium was determined by comparing their optical density to the standard curve.

#### Self-renewal assay.

Single cell suspensions of IPF and control MPCs (5,000 cells) were incorporated into methylcellulose gels and maintained in E8 medium (StemCell Technologies, Cambridge, MA) (37°C, 5% CO_2_; 1 wk). The capacity of cells to form colonies was quantified after addition of human recombinant IL-8 (Peprotech; Rocky Hills, NJ). Enumeration of colonies was performed microscopically.

#### Proliferation assay.

In some experiments, 5 × 10^4^ IPF and control MPC progeny were seeded on 96-well plates containing DMEM + 1% FBS and recombinant IL-8 and cultured for 16 h. Proliferation was quantified using the MTT Proliferation Assay per the manufacturer’s instructions (Roche).

#### Migration assay.

The effect of recombinant IL-8 (PeproTech, Rocky Hill, NJ) on the migration of IPF and control MPC progeny was examined using the QCM Chemotaxis Cell Migration Assay Kit per the manufacturer’s instructions (EMD Millipore, Billerica MA). Briefly, 2 × 10^4^ IPF MPC progeny were added to the top of a filter insert (8-µm pore size) containing 100 µl of serum free DMEM in a 96-well plate. One-hundred and fifty microliters of DMEM containing recombinant IL-8 were added to the lower chamber, and the cells were allowed to migrate for 16 h. The number of migrating cells were quantified using CyQuant GR dye and a fluorescent plate reader (SpectraMax M3).

#### Macrophage migration assay.

We utilized the human monocyte cell line U937 to generate human macrophages for the macrophage migration assay. U937 cells were maintained with RPMI 1640 containing 10% FBS. To induce monocyte differentiation, the U937 cells were treated with 20 nM phorbol myristate acetate in RPMI 1640 medium for 16 h. The media were then replaced with fresh media, and the cells were cultured for an additional 3 days to permit monocyte differentiation to macrophages. We then examined the effect of IPF MPC progeny-derived conditioned medium on macrophage migration using the QCM Chemotaxis Cell Migration Assay Kit. Briefly, 2 × 10^4^ macrophages were added to the top of a filter insert (8-µm pore size) containing 100 µl of serum free DMEM in a 96-well plate. One-hundred and fifty microliters of IPF MPC progeny-derived conditioned medium were added to the lower well. In some experiments, the CXCR1/2 inhibitor reparixin (100 µM) or the CXCR2 inhibitor SB225002 (1 µM) or DMSO control was added to the IPF MPC-progeny-derived conditioned medium. The migration assay was conducted for 16 h. The number of cells migrating to the lower side of the filter insert was quantified using CyQuant GR dye and a fluorescent plate reader.

#### Immunohistochemistry and in situ hybridization.

Immunohistochemistry was performed on 4-µm paraffin-embedded serial sectioned IPF and control lung tissue and mounted on polylysine-coated slides. The sections were deparaffinized in xylene, rehydrated through a graded Methanol series, quenched with 0.3% hydrogen peroxide in methanol, and immersed in a 98°C water bath for 30 min in citrate buffer (pH 6·0) for antigen retrieval. Sections were placed in 5% normal horse serum (Jackson Immunoresearch, West Grove, PA) to block nonspecific binding of secondary antibodies. Endogenous avidin and biotin binding sites were blocked by sequential incubation for 15 min each with an Avidin/Biotin Blocking Kit (Vector Laboratories, Burlingame, CA) and incubated overnight (18–20 h, 4°C) with the following primary antibodies: S100A4 (1:8000; Cat. No. 07-2274, Millipore, Billerica, MA), CD163 (1:2,000; Cat. No. ab182422; Abcam, Cambridge, MA), CXCR1 (1:2,000; Cat. No. ab137351; Abcam), CXCR2 (1:200; Cat. No. MAB331100; R&D Systems, Minneapolis, MN), and SSEA4 (1:100; Clone MC-813-70; Cat. No. 330402; Biolegend, San Diego, CA). Sections were rinsed with PBS and placed in biotinylated horse anti-Mouse IgG secondary antibody (1:500) for 30 min at room temperature, followed by R.T.U. Horseradish Peroxidase Streptavidin Complex (Vector Laboratories) for 30 min. Specific antibody binding was detected using a 3,3′-diaminobenzidine peroxidase kit (DAB; Vector). Sections were counterstained with hematoxylin (Invitrogen, Frederick, MD) for 2 min, and PBS was applied for 30 min. Specimens were coverslipped with a Prolong Antifade Kit (Invitrogen/Molecular Probes) and stored overnight at room temperature without light before image analysis.

To detect cells expressing IL-8 RNA, in situ hybridization was performed on IPF lung tissue specimens using RNAscope 2.5 Reagent Kit according to the manufacturer’s instructions (Advanced Cell Diagnostics, Hayward, CA).

#### RNA sequencing data.

We used previously generated RNA sequencing data (40). The complete data sets and protocols are deposited in data repository Gene Expression Omnibus (Accession No. GSE97038).

#### Data analysis.

Comparisons of data among experiments were performed with the two-tailed Student’s *t*-test. Experiments were independently replicated a minimum of three times. Data are expressed as means ± SD. *P* < 0.05 was considered significant.

## RESULTS

### 

#### IL-8 expression is increased in IPF MPCs and their progeny.

**Fig. 1.**
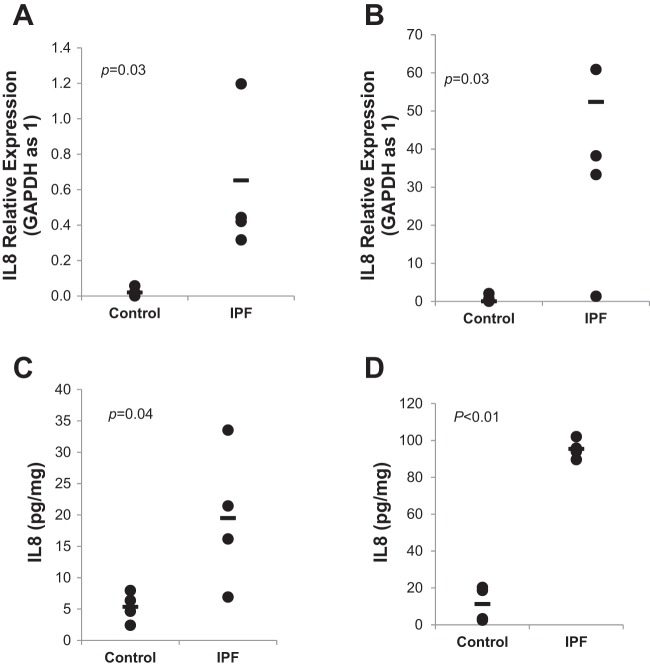
IL-8 expression is increased in idiopathic pulmonary fibrosis (IPF) mesenchymal progenitor cells (MPCs). *A* and *B*: IL-8 mRNA expression in IPF and control MPCs (*A*) and IPF and control MPC progeny (*B*). Shown are relative expression levels of each mRNA by quantitative (Q)-PCR. Data shown represents the mean mRNA levels in IPF MPCs and IPF MPC progeny and control MPCs and control MPC progeny (derived from 4 cell lines each). *C* and *D*. IL-8 protein levels were quantified in cell lysates of IPF and control MPCs (*C*) and IPF and control MPC progeny (*D*) by ELISA (*n* = 4 cell lines each). Data are expressed as means ± SE. *P* values were determined by two-tailed Student’s *t*-test.

Our previous gene expression profiling studies demonstrated that IPF MPCs display a distinct IPF MPC transcriptome that distinguishes them from control MPCs (40). Our transcriptomic analysis demonstrated a striking coregulation of genes between IPF MPCs and IPF MPC progeny, with many genes either upregulated or downregulated in both (40). Among the coregulated genes that distinguish IPF from control, we identified IL-8 as a candidate regulator of IPF MPC fibrogenicity (40). To test the validity of this result, we directly quantified IL-8 mRNA expression in IPF and control MPCs and their progeny. Q-PCR verified the transcriptomic analysis demonstrating an ~20-fold higher level of IL-8 expression in IPF MPCs compared with control MPCs (Fig. 1*A*) and an ~25-fold difference in IL-8 mRNA expression between IPF MPC progeny and control MPC progeny (Fig. 1*B*). It is important to note that we found markedly higher levels of IL-8 in IPF MPC progeny compared with IPF MPCs. Q-PCR revealed an ~80-fold increase in IL-8 mRNA expression in IPF MPC progeny compared with IPF MPCs. This indicates that as IPF MPCs differentiate to IPF MPC progeny over 21 days, this is accompanied by a marked increase in IL-8 gene expression (see Fig. 1, *A* and *B*). In contrast, there was only minimal difference in IL-8 gene expression between control MPCs and their *day 21* progeny. This is consistent with our previously published results demonstrating that IPF MPCs differentiate to progeny that display a distinct fibrogenic phenotype that is characteristic of IPF fibroblasts and is different from the phenotype of control fibroblasts derived from control MPCs (42).

We next examined IL-8 protein expression. Consistent with the mRNA data, there was coregulation of IL-8 protein expression in IPF MPCs and IPF MPC progeny with fourfold higher levels of IL-8 in IPF MPCs compared with control MPCs and approximately ninefold higher levels of IL-8 protein in IPF MPC progeny compared with control MPC progeny (Fig. 1, *C* and *D*). Consistent with the mRNA data, IL-8 protein levels were approximately fivefold higher in IPC MPC progeny compared with IPF MPCs; however, there was only a modest difference in IL-8 protein levels between control MPCs and their progeny. These data indicate that IL-8 expression is higher in IPF MPCs and their progeny compared with their control counterparts and that IL-8 protein levels increase as IPF MPCs proliferate and differentiate into their progeny.

#### IPF MPCs and IPF MPC progeny actively secrete IL-8.

**Fig. 2.**
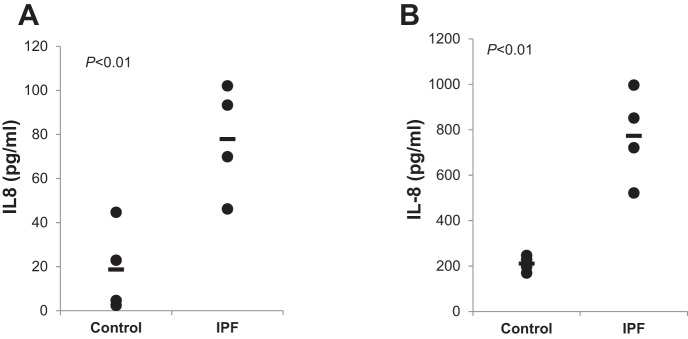
IPF MPCs and IPF MPC progeny actively secrete IL-8. *A* and *B*: IPF and control MPCs (*A*) or IPF and control MPC progeny (*B*) were seeded on tissue culture dishes in fresh DMEM and cultured for 2 days. The medium was collected, and IL-8 protein levels were quantified in cell culture medium by ELISA. Data shown represent the mean protein levels in IPF MPCs and IPF MPC progeny and control MPCs and control MPC progeny (derived from 4 cell lines each). Data are expressed as means ± SE. *P* values were determined by two-tailed Student’s *t*-test.

A variety of cells, both normal and malignant, secrete IL-8 (11, 20, 21, 36, 37, 43). To determine whether IPF MPCs secrete IL-8, we measured IL-8 protein levels in IPF MPC culture medium by ELISA and compared it to control MPCs. When cultured for 2 days, IL-8 protein levels in IPF MPC-derived culture medium were approximately fourfold higher compared with control MPC-derived culture medium (Fig. 2*A*). Similarly, IL-8 levels were fourfold higher in the culture medium derived from IPF MPC progeny compared with the culture medium derived from control MPC progeny. At both the mRNA and protein levels, we found a striking increase in IL-8 as IPF MPCs proliferate and differentiate into their progeny. Consistent with this we found a marked increase in IL-8 secretion in IPF MPC progeny compared with IPF MPCs. IL-8 secretion was ~10-fold higher in IPF MPC progeny compared with IPF MPCs. These data indicate that IPF MPCs actively secrete IL-8 which markedly increases as the cells differentiate.

We also examined whether IPF MPCs or their progeny express and secrete other CXCR1/2 ligands in addition to IL-8. We first examined IPF MPC expression of CXCL1, CXCL3, CXCL5, and CXCL6. IPF MPCs expressed CXCL1 but displayed low expression of CXCL3, CXCL5, or CXCL6 (data not shown). Therefore, we examined whether IPF MPCs or their progeny secrete CXCL1. Both IPF and control MPCs secreted low levels of CXCl-1 (~3 pg/ml and ~2.5 pg/ml, respectively). In addition, IPF and control MPC progeny also secreted relatively low levels of CXCL-1 (~6 and ~4.5 pg/ml. respectively). Compared with IL-8, IPF MPCs secreted ~10-fold lower levels of CXCL-1 and IPF MPC progeny secreted ~100-fold less CXCL-1. Thus IL-8 is a major CXCR1/2 ligand expressed and secreted by IPF MPCs and their progeny.

#### Role of IL-8/CXCR1 axis in promoting IPF MPC self-renewal.

**Fig. 3.**
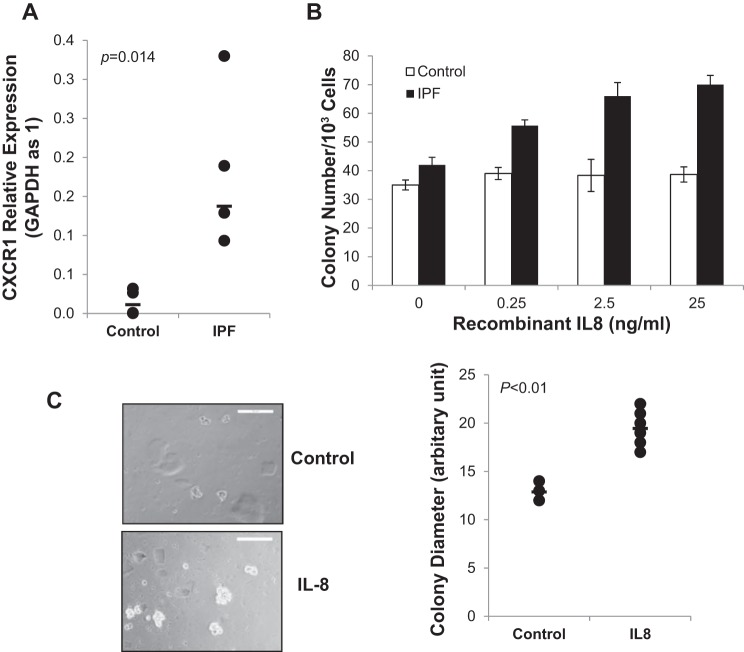
IL-8 promotes IPF MPC self-renewal. *A*: IPF and control MPC (*n* = 4 cell lines each) CXCR1 mRNA expression levels were quantified by Q-PCR. *B* and *C*: to analyze the effect of IL-8 on IPF and control MPC self-renewal, 5,000 cells in a single cell suspension were seeded per well in 24-well dishes containing methylcellulose and recombinant IL-8 as indicated. The cells were cultured for 7 days. *B*: colony number per 5,000 cells was quantified microscopically (*n* = 3). *C*: graph depicting colony size and phase contrast image of IPF colony formation. Data are expressed as means ± SE. *P* values were determined by two-tailed Student’s *t*-test.

Ligation of the IL-8 receptor CXCR1 by IL-8 in breast cancer cells increases cancer stem cell self-renewal thereby expanding the pool of cancer stem cells (9). Since IPF MPCs actively secrete IL-8, we sought to determine whether IL-8 functioned in an autocrine manner to stimulate IPF MPC self-renewal. We first analyzed CXCR1 expression in IPF and control MPCs. CXCR1 mRNA expression was elevated approximately fivefold in IPF MPCs compared with control (Fig. 3*A*).

To determine the effect of IL-8 on MPC self-renewal, we treated IPF and control MPCs with human recombinant IL-8 and determined its effect on self-renewal by quantifying colony formation. Recombinant IL-8 augmented IPF MPC colony formation in a dose-dependent manner (Fig. 3*B*). Recombinant IL-8 increased both colony number and size (Fig. 3*C*). In contrast, there was minimal effect of recombinant IL-8 on control MPC colony formation. These data indicate that IL-8 functions in an autocrine manner to promote IPF MPC self-renewal.

#### IL-8 promotes the proliferation and motility of IPF MPC progeny.

**Fig. 4.**
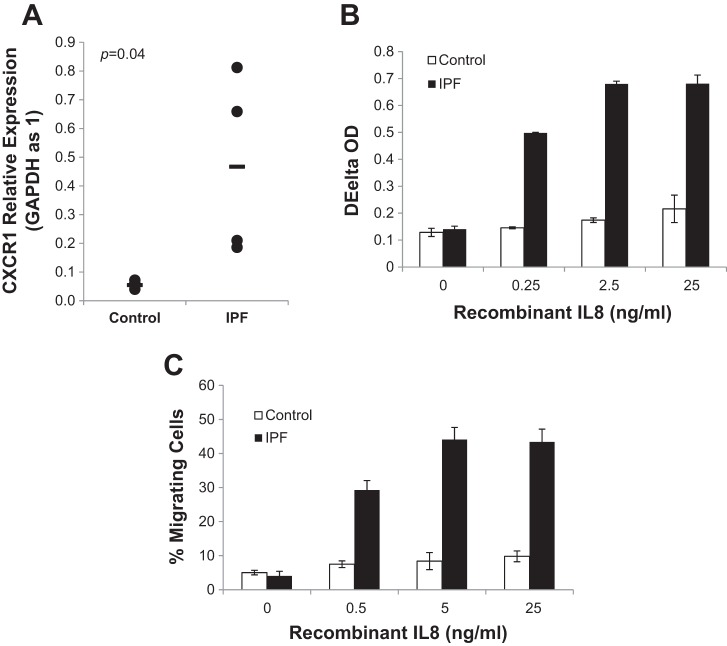
IL-8 promotes the proliferation and motility of IPF MPC progeny. *A*: CXCR1 mRNA expression levels were quantified in IPF and control MPC progeny (*n* = 4 cell lines each) by Q-PCR. *B*: shown is proliferation of IPF and control MPC progeny in response to recombinant IL-8. Cell number was quantified using the MTT Proliferation Assay Kit (*n* = 3). OD, optical density. *C*: shown is the migration of IPF and control MPC progeny in response to recombinant IL-8. Migration was quantified using the QCM Chemotaxis Cell Migration Assay Kit (*n* = 3). Data are expressed as means ± SE. *P* values were determined by two-tailed Student’s *t*-test.

Our mRNA and protein analyses revealed a marked increase in IL-8 expression as IPF MPCs differentiated to their progeny, suggesting an important role for IL-8 in regulating the function of IPF MPC progeny. We first examined CXCR1 expression in IPF MPC progeny and found a ~20-fold higher level of CXCR1 expression compared with control MPC progeny (Fig. 4*A*). In accord with this, recombinant IL-8 increased the proliferation of IPF MPC progeny in a dose-dependent manner (Fig. 4*B*), whereas the proliferation of control fibroblasts treated with recombinant IL-8 was not appreciably altered.

The IL-8/CXCR1 axis has also been reported to regulate cancer stem cell motility and invasion (38). Using a chemotaxis assay and recombinant IL-8 as a chemoattractant, we found that recombinant IL-8 stimulated the migration of IPF MPC progeny in a dose-dependent manner (Fig. 4*C*) but did not significantly affect the motility of control MPC progeny. Taken together, these results indicate that IL-8 functions in an autocrine manner to promote the proliferation and motility of IPF MPC progeny. Our data suggest that signaling through the IL-8/CXCR1/2 axis may facilitate the proliferation and invasion of IPF MPC progeny into adjacent alveolar structures during fibrotic progression.

#### Paracrine function of IL-8 secreted by IPF MPCs.

**Fig. 5.**
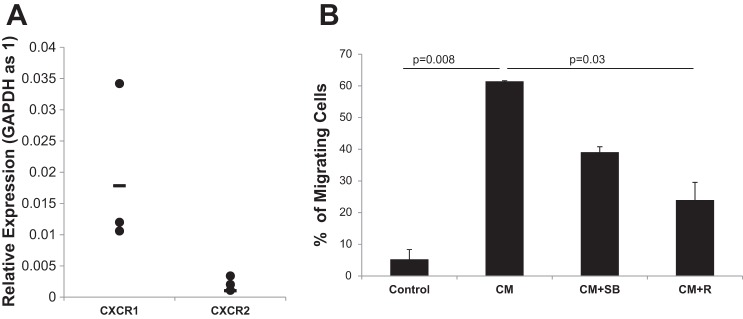
Paracrine function of IL-8 secreted by IPF MPCs. *A*: CXCR1 and CXCR2 mRNA expression in macrophages. *B*: macrophage migration in response to fresh medium (control), IPF MPC progeny-derived conditioned medium containing DMSO (CM), IPF MPC progeny-derived conditioned medium containing the CXCR2 inhibitor SB332235 (1 µM) (CM + SB), and IPF MPC progeny-derived conditioned medium containing the CXCR1/2 inhibitor reparixin (100 µM) (CM + R) were analyzed using the QCM Chemotaxis Cell Migration Assay Kit (*n* = 3). Data are expressed as means ± SE. *P* values were determined by two-tailed Student’s *t*-test.

IL-8 serves as a chemoattractant for immune cells including macrophages and IPF MPCs and their progeny secrete IL-8. Since IPF MPC progeny secrete ~10-fold higher amounts of IL-8 compared with IPF MPCs, we examined whether IPF MPC progeny-derived conditioned medium affected macrophage migration. We first examined CXCR1 and CXCR2 expression by the macrophages. Both CXCR1 and CXCR2 were expressed by macrophages, with higher expression of CXCR1 compared with CXCR2 (Fig. 5*A*). We found that the conditioned medium stimulated macrophage migration 95% (Fig. 5*B*). The effect of the conditioned medium on macrophage migration was inhibited 60% by the CXCR1/2 inhibitor reparixin and 35% by the CXCR2 inhibitor SB225002, indicating that IL-8 in the conditioned medium was a major contributor to the enhanced macrophage migration. These data raise the possibility that IL-8 secreted by IPF MPCs and their progeny may promote the recruitment of macrophages to the active fibrotic front of the fibroblastic focus.

#### IPF MPCs codistribute with macrophages in the peripheral region of the fibroblastic focus.

**Fig. 6.**
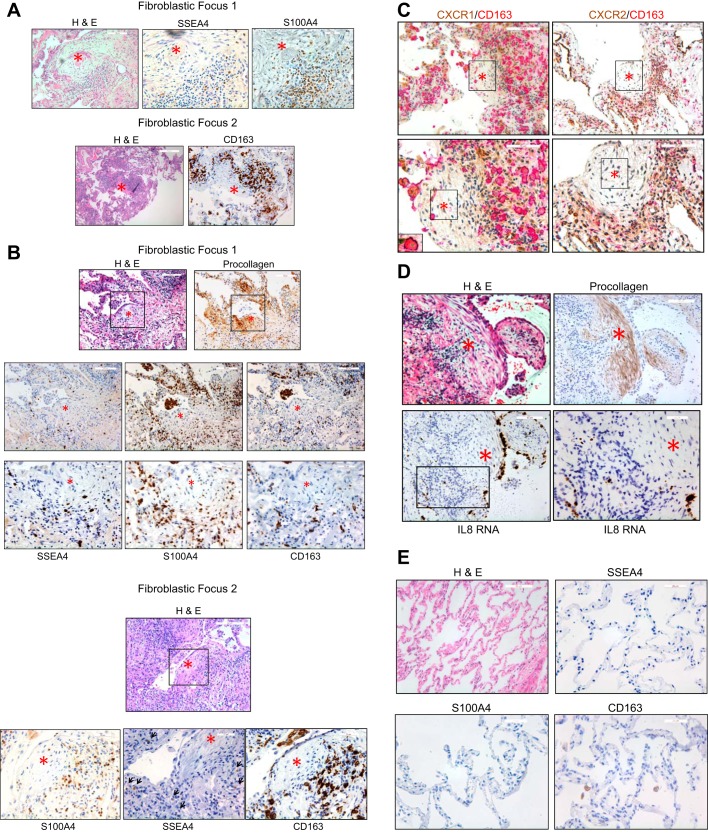
IPF MPCs colocalize with macrophages in the peripheral region of the fibroblastic focus. Immunohistochemical (IHC) was performed on human IPF lung tissue (*n* = 10 IPF patient specimens). *A*: shown are 2 fibroblastic foci. Hematoxylin and eosin (H&E) staining was used to identify the fibroblastic focus and IHC was performed using CD163 (macrophage marker) and SSEA4 and S100A4 (MPC markers) antibodies to assess distribution of macrophages and IPF MPCs in the focus. Asterisk denotes focus core. Bar = 50 μm (*left*) and 20 μm (*middle* and *right*). *B*: shown are serial 4-μm sections stained for H&E, procollagen, SSEA4, S100A4, and CD163. Shown are two fibroblastic foci. Procollagen expression was used to delineate the myofibroblast-rich focus core. IHC analysis of the foci demonstrated S100A4- and SSEA4-positive cells at the periphery of the focus. CD163-positive cells codistributed with SSEA4- and S100A4-expressing cells at the focus perimeter. Asterisk denotes focus core. Fibroblastic Focus 1: bar = 50 μm (*top* and *middle*) and 20 μm (*bottom*); Fibroblastic Focus 2: bar = 50 μm (*top*) and 20 μm (*bottom*). *C*: IHC analysis of IPF lung tissue for CXCR1 and CXCR2 expression and CD163. *Left*: dual staining for CXCR1 (brown) and CD163 (red). Shown in the *inset* is a CD163-positive macrophage (red) with CXCR1 labeling (brown) on the perimeter of the cell. *Right*: dual staining for CXCR2 (brown) and CD163 (red). Bar = 100 μm (*top*) and 50 μm (*bottom*). Asterisk denotes focus core. *D*: in situ hybridization for IL-8 was performed on IPF lung tissue. Bar = 50 μm (*top* and *bottom left*) and 20 μm (*bottom right*). *E*: H&E staining and IHC was performed on human control lung tissue (*n* = 3 control patient specimens) using antibodies to S100A4, SSEA4, and CD163. Bar = 50 μm (H&E) and 20 μm (IHC).

While the precise role of innate and adaptive immunity in IPF pathogenesis is controversial, a number of studies have documented the presence of immunomodulatory cells in regions of active disease in IPF lung tissue (30–32). We recently reported that the fibroblastic focus is a polarized structure with IPF MPCs and their early generation transit amplifying daughter cells located at the periphery of the focus in an interface region between the focus core containing α-smooth muscle actin-expressing myofibroblasts and more normal contiguous alveolar structures (42). This interface region is a highly cellular area containing numerous Ki67-expressing cells indicating that this region is an active cellular front. Since IPF MPCs and IPF MPC progeny actively secrete IL-8 and recruit macrophages in vitro, this suggested to us that IPF MPCs may codistribute with tissue macrophages at the active cellular front at the perimeter of the fibroblastic focus. We performed immunohistochemical analysis on IPF lung tissue to examine the distribution of IPF MPCs with tissue macrophages. We used SSEA4 expression as a marker of IPF MPCs (40, 42) and CD163 expression as a macrophage marker (1, 2, 12, 22, 24). We have recently discovered that IPF MPCs and their early generation progeny highly express S100A4, a calcium-binding protein also expressed by cancer stem cells, as well as some immune cells (42). Therefore we also examined S100A4 expression in relation to CD163. Consistent with our prior findings (42), our analysis revealed SSEA4- and S100A4-expressing cells at the periphery of the fibroblastic focus in a highly cellular interface region between the myofibroblast core and less involved alveolar structures (Fig. 6*A*, *top)*. Numerous CD163-positive cells were present on the periphery of the focus in a similar distribution as IPF MPCs (Fig. 6*A*, *bottom*). To confirm this, we analyzed serial sections of fibroblastic foci. Serially sectioned fibroblastic foci demonstrated procollagen-expressing cells in the myofibroblast-rich focus core and SSEA4- and S100A4-expressing cells codistributing with CD163-positive cells on the periphery of the focus (Fig. 6*B*). The CD163 staining indicated that many of the cells infiltrating in this region were “activated” macrophages (1, 2, 12, 22). Since IPF MPCs and their progeny as well as macrophages express the IL-8 receptors CXCR1 and CXCR2, we examined CXCR1 and CXCR2 expression in IPF lung tissue. CXCR1- and CXCR2-immunoreactive cells codistributed with CD163-expressing macrophages in the active cellular front located on the perimeter of the fibroblastic focus (Fig. 6*C*). This is in the same distribution as IL-8-secreting IPF MPCs. Individual macrophages immunoreactive for both CXCR1 and CD163 were readily apparent (see Fig. 6*C*, *bottom left inset*). Consistent with the immunohistochemical findings, in situ hybridization revealed cells staining positive for IL-8 on the periphery of the fibroblastic focus (Fig. 6*D*). While cells within the focus core were devoid of IL-8 staining, epithelial cells overlying the procollagen-positive focus core stained positive for IL-8. In contrast, there was a paucity of S100A4- and SSEA4-expressing cells in normal alveolar structures in patient control lung tissue specimens (Fig. 6*E*). Occasional CD163-positive alveolar macrophages were present in the airspaces of control lung tissue (Fig. 6*E*). No immunoreactivity was seen in IPF or control lung tissue when using isotype control antibodies (data not shown). The colocalization of IPF MPCs and macrophages at the perimeter of the focus raise the possibility that IPF MPCs and their progeny may recruit macrophages to the active cellular front of the IPF fibroblastic focus.

## DISCUSSION

The mechanisms underlying the relentless progression of fibrosis in IPF remain incompletely understood. We previously identified pathological MPCs as a source of the activated myofibroblasts mediating fibrotic destruction in IPF (40, 42). The concept that fibrotic progression is driven by a fibrogenic MPC has fundamental implications for fibrosis biology and treatment. This work represents a paradigm shift directing IPF experimental therapeutics toward a specific, fibrogenic progenitor cell (sub)population rather that the activated myofibroblast population as a whole; analogous to what has occurred in cancer biology with the discovery of cancer stem cells/tumor-initiating cells as the cells that must be targeted to achieve a cure. Nevertheless, despite this discovery, the mechanisms governing IPF MPC fibrogenicity are incompletely defined. In this report, we show that IPF MPCs and their progeny secrete markedly increased amounts of IL-8 compared with control cells, which can function in an autocrine and paracrine manner to increase IPF mesenchymal cell population size, motility, and macrophage recruitment.

Elevated IL-8 levels are present in the bronchoalveolar lavage fluid, sputum, and serum of IPF patients (7, 13, 33, 44, 45). Alveolar macrophages isolated from IPF patients have elevated IL-8 mRNA expression, which correlates with IL-8 protein levels in bronchoalveolar lavage fluid and neutrophil numbers in lavage fluid (8, 25). Importantly, elevated levels of IL-8 correlate with disease activity and have been shown to predict mortality in IPF (7, 45). Notwithstanding these reports, the precise role of IL-8 and immunomodulatory cells in IPF remains unclear (6). This is illustrated by studies showing that overexpression of macrophage inflammatory protein 2, a rodent chemokine closely resembling IL-8, while causing pneumonitis did not result in fibrosis, suggesting that IL-8 may play an indirect role in fibrotic progression (18). Recent reports in the cancer literature indicate that IL-8, signaling through CXCR1/2, promotes cancer stem cell self-renewal, stimulating mammosphere colony formation in breast cancer stem cells (9, 38). Here we show that IPF MPCs display increased expression of IL-8 compared with controls and that IL-8 levels markedly increase as IPF MPCs differentiate in culture. We demonstrate that both IPF MPCs and their progeny secrete large amounts of IL-8 and express the IL-8 cognate receptor CXCR1. Our data indicate that IL-8 functions in an autocrine manner and its effects are dependent on the differentiation state of the MPCs. In IPF MPCs, IL-8 promoted self-renewal, as demonstrated by both by increased colony size as well as number, indicating that IL-8 stimulated IPF MPC division giving rise to additional MPCs thereby expanding the IPF MPC population. In contrast, in the differentiated IPF MPC progeny, IL-8 promoted both proliferation and motility. Our data also demonstrate that IL-8 functions in a paracrine manner. Both IPF MPCs and their progeny secrete IL-8; however, the amount of IL-8 secreted by the progeny is ~10-fold higher compared with progenitors, indicating that as the progenitors differentiate they secrete more IL-8. We demonstrate that IL-8 secreted by IPF MPC progeny stimulates macrophage migration, suggesting that IPF MPCs and IPF MPC progeny promote macrophage recruitment. Together, these data indicate that IL-8 functions in an autocrine and paracrine fashion to regulate multiple fibrogenic functions of IPF MPCs and their progeny.

In prior immunohistochemical studies examining fibroblastic foci, we found that fibroblastic foci, located at the advancing fibrotic front, are polarized structures with activated myofibroblasts in the focus core and IPF MPCs and their early generation daughter cells located in the focus periphery in an interface region between the myofibroblastic core and adjacent intact alveolar structures (42). Interestingly, we found that the interface region at the periphery of the focus is a highly cellular region containing proliferating cells indicating that it is an active cellular front. These findings support the concept that there may be growth of the fibroblastic focus as the lesion evolves with infiltration of the active fibrotic front into contiguous alveolar structures (42). In a continued effort to define the polarized fibroblastic focus and the active fibrotic front, here we demonstrate that numerous activated macrophages are interspersed with IPF MPCs and their early generation progeny in the active cellular front on the periphery of the fibroblastic focus. These data together with our in vitro data showing that the conditioned media derived from IPF MPC progeny promotes macrophage motility in an IL-8-dependent manner, suggest that IPF MPCs and their early generation progeny actively synthesize and secrete IL-8 thereby recruiting immunomodulatory cells to sites of active disease. Our in situ hybridization analysis of IPF lung tissue supports this concept. Scattered IL-8-positive cells were present on the focus perimeter, in the same location as IPF MPCs and CD163-positive activated macrophages. While the precise identity of these IL-8-expressing cells is currently unclear, our data suggest that at least some are IPF MPCs and IPF MPC progeny. Interestingly, while the focus core was devoid of IL-8-expressing cells, epithelial cells overlying the focus core strongly expressed IL-8. Since IPF bronchoalveolar lavage fluid contains IL-8, it raises the possibility that epithelial cells may release IL-8 recruiting alveolar macrophages to the affected region. Consistent with this concept, a recent study found that monocyte-derived alveolar macrophages were recruited to the lung in the aftermath of bleomycin-induced pulmonary injury and were important in driving the development of lung fibrosis (26). Once recruited to sites of active disease, activated macrophages can release growth factors that promote the proliferation of mesenchymal cells (5). In support of these concepts, recent reports exploring properties of the invasive front of cancer tissue demonstrated the presence of large numbers of CD163-positive tumor-associated infiltrating macrophages at the invasive front, which codistributed with cells with high transforming growth factor-β activity (1, 12). In conclusion, these data suggest that cross talk between fibrogenic mesenchymal progenitor cells and activated macrophages in the active fibrotic front of the fibroblastic focus promote the expansion of the mesenchymal cell population at the fibrotic front and subsequent infiltration of the front into adjacent normal alveolar structures enlarging the fibroblastic focus and driving fibrotic progression. These data suggest that to arrest fibrotic progression treatment strategies targeting IPF MPCs and their progeny as well as activated macrophages within the active fibrotic front will be required.

## GRANTS

This work was supported by National Heart, Lung, and Blood Institute Grants R01-HL-125227 (to C. A. Henke) and R01-HL-125236 (to P.B. Bitterman) and funds provided by the Witowski and O’Brien families.

## DISCLOSURES

No conflicts of interest, financial or otherwise, are declared by the authors.

## AUTHOR CONTRIBUTIONS

L.Y. and C.A.H. conceived and designed research; L.Y., J.H., A.J.G., H.X., K.S., and A.B. performed experiments; L.Y., J.H., A.J.G., H.X., K.S., and A.B. analyzed data; L.Y., J.H., A.J.G., H.X., K.S., A.B., and C.A.H. interpreted results of experiments; L.Y. prepared figures; L.Y., P.B.B., and C.A.H. drafted manuscript; L.Y., J.H., A.J.G., H.X., K.S., A.B., P.B.B., and C.A.H. edited and revised manuscript; L.Y., J.H., A.J.G., H.X., K.S., A.B., P.B.B., and C.A.H. approved final version of manuscript.
